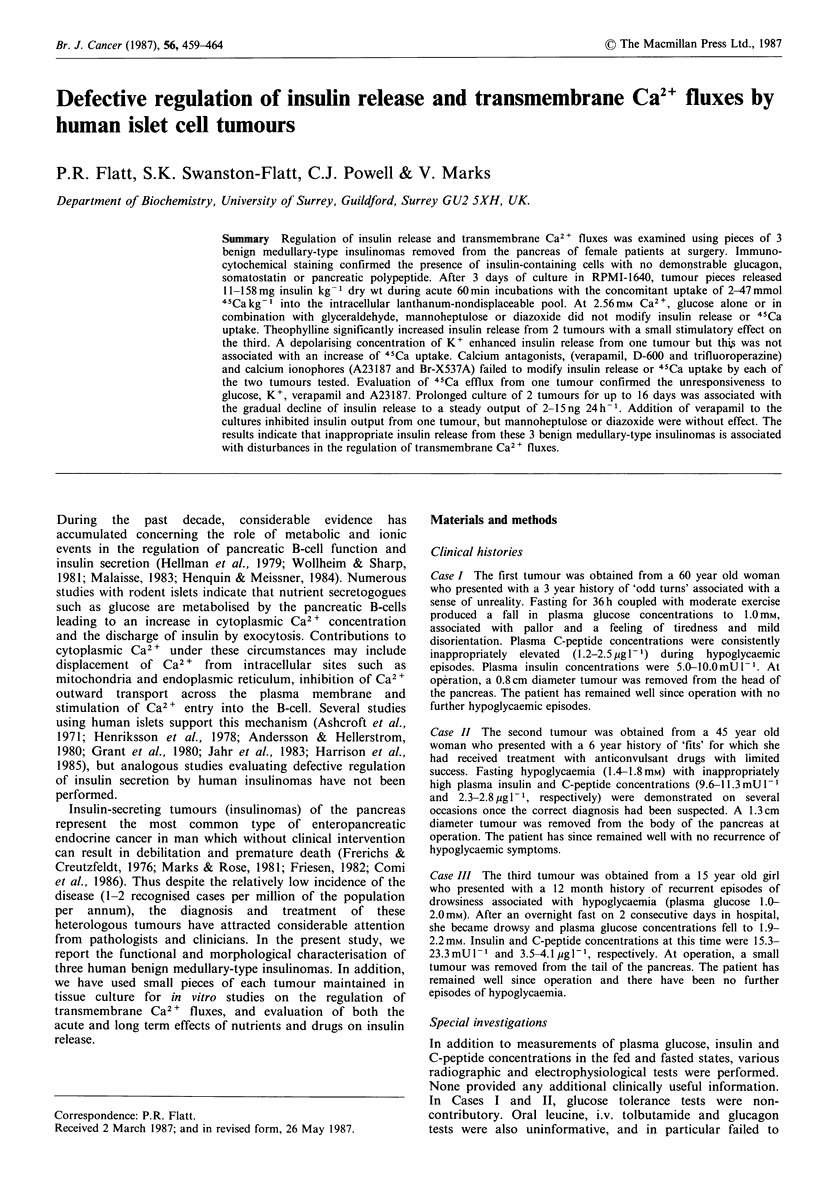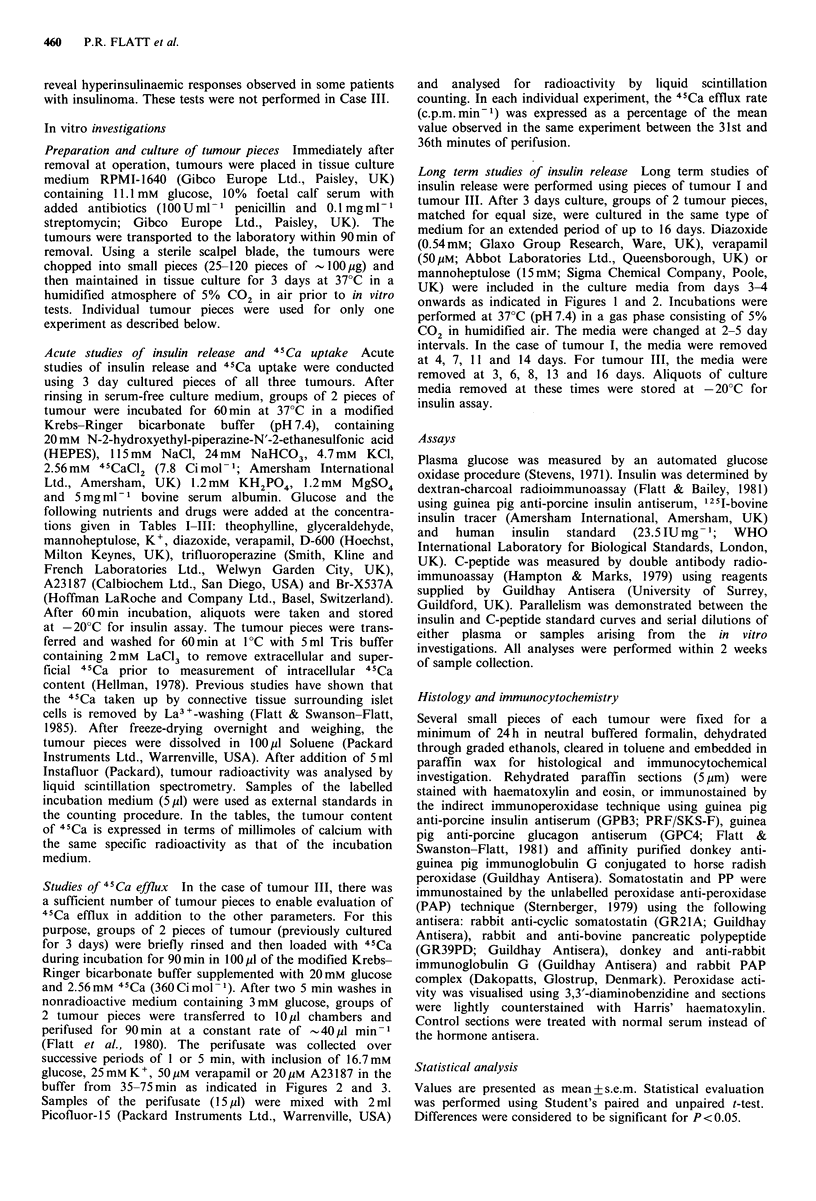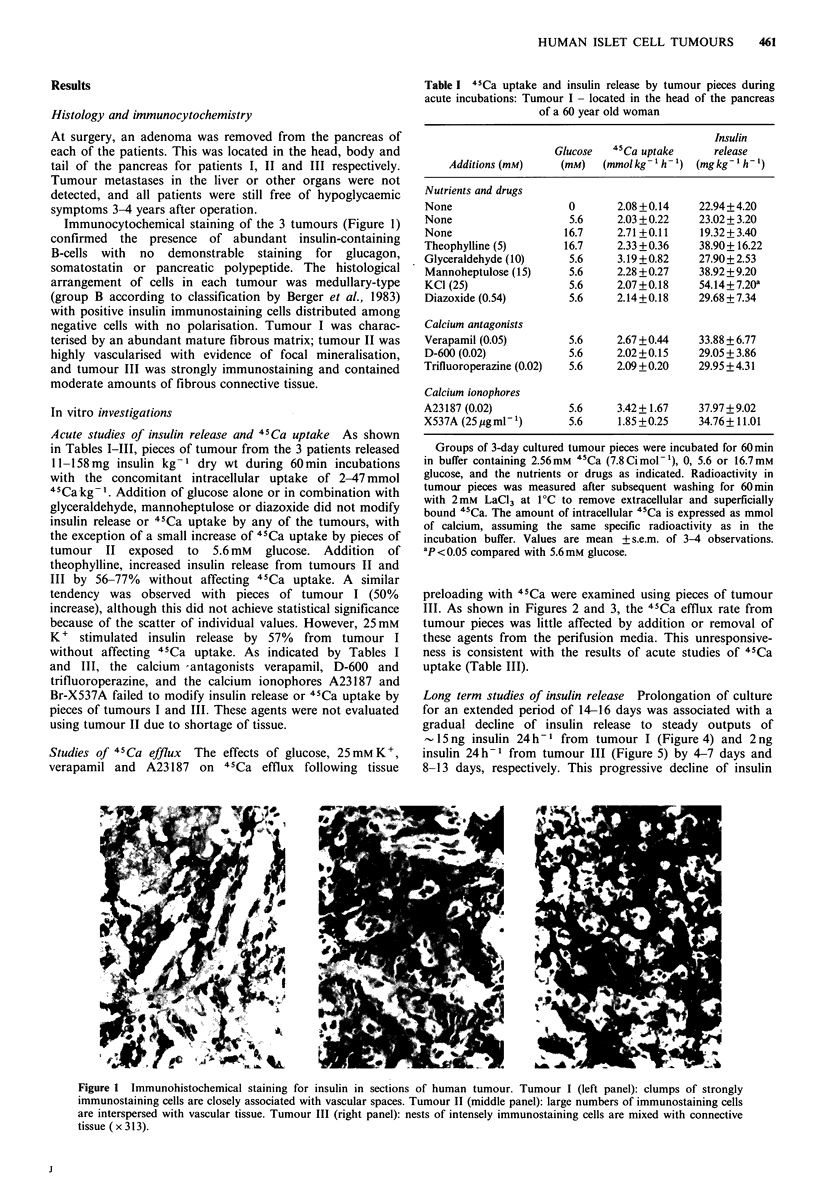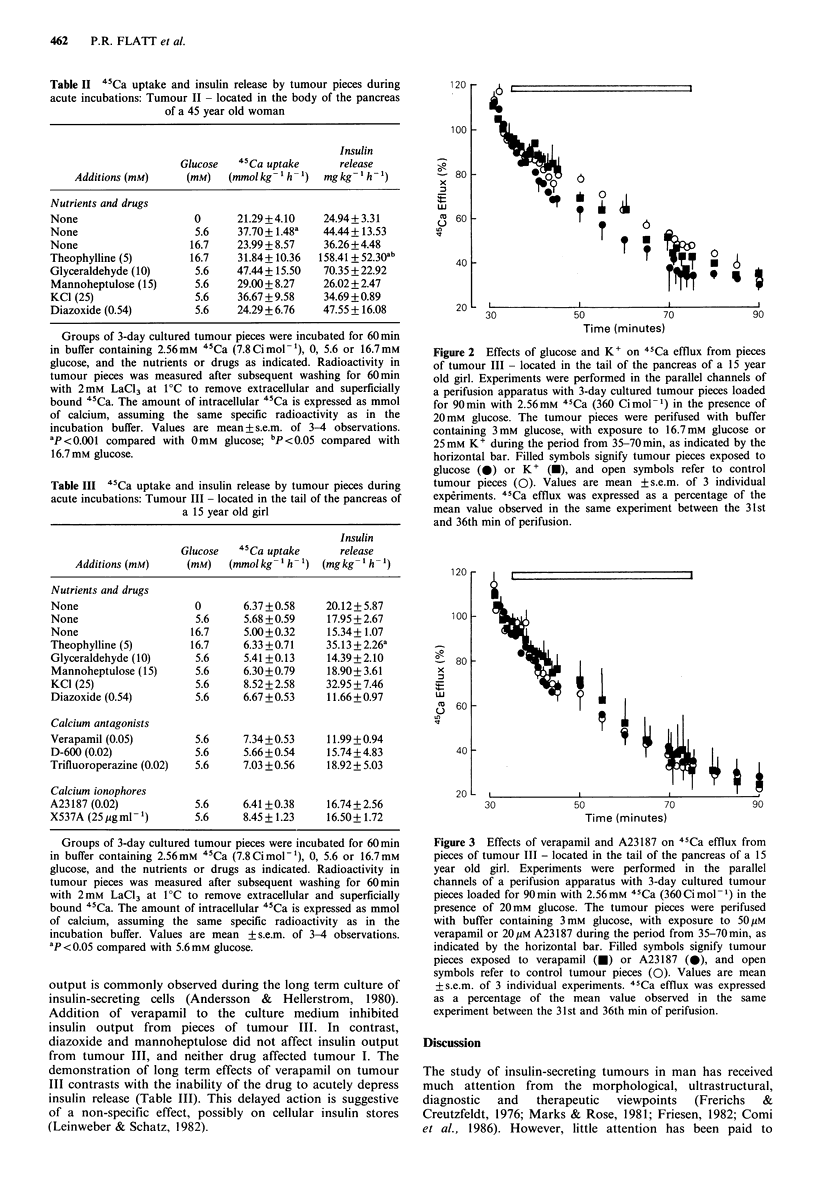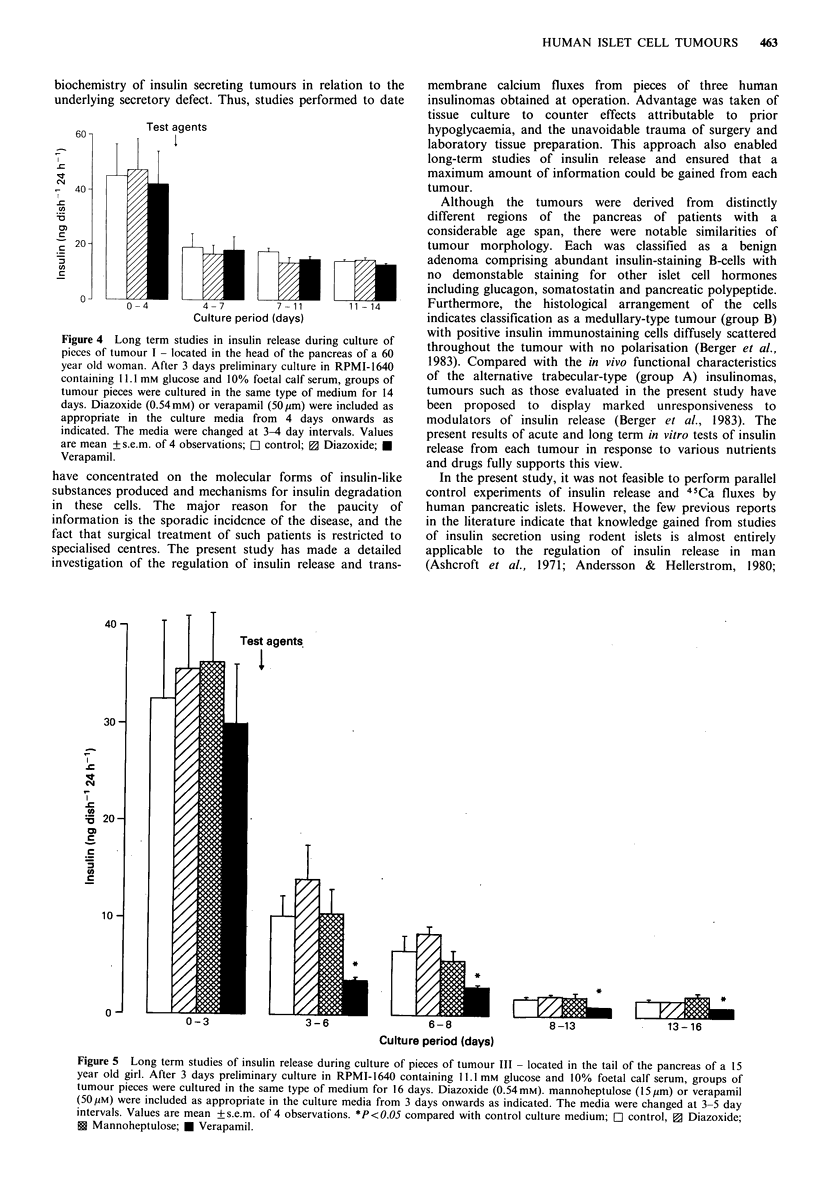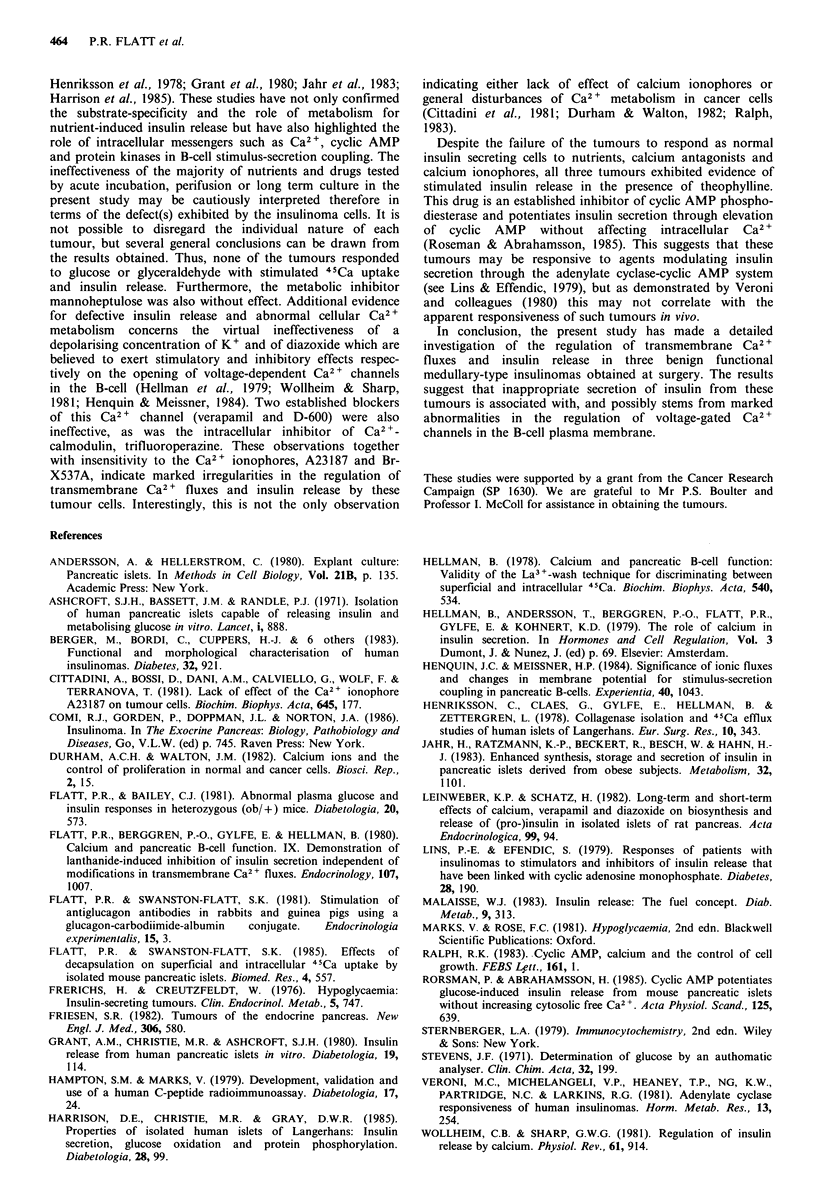# Defective regulation of insulin release and transmembrane Ca2+ fluxes by human islet cell tumours.

**DOI:** 10.1038/bjc.1987.224

**Published:** 1987-10

**Authors:** P. R. Flatt, S. K. Swanston-Flatt, C. J. Powell, V. Marks

**Affiliations:** Department of Biochemistry, University of Surrey, Guildford, UK.

## Abstract

**Images:**


Br. J. Cancer (1987), 56, 459–464 © The Macmillan Press Ltd., 1987

### Defective regulation of insulin release and transmembrane $Ca^{2+}$ fluxes by human islet cell tumours

P.R. Flatt, S.K. Swanston-Flatt, C.J. Powell & V. Marks

*Department of Biochemistry, University of Surrey, Guildford, Surrey GU2 5XH, UK.*

**Summary** Regulation of insulin release and transmembrane $Ca^{2+}$ fluxes was examined using pieces of 3 benign medullary-type insulinomas removed from the pancreas of female patients at surgery. Immunocytochemical staining confirmed the presence of insulin-containing cells with no demonstrable glucagon, somatostatin or pancreatic polypeptide. After 3 days of culture in RPMI-1640, tumour pieces released 11–158 mg insulin $kg^{-1}$ dry wt during acute 60 min incubations with the concomitant uptake of 2–47 mmol $^{45}Ca\,kg^{-1}$ into the intracellular lanthanum-nondisplaceable pool. At 2.56 mM $Ca^{2+}$, glucose alone or in combination with glyceraldehyde, mannoheptulose or diazoxide did not modify insulin release or $^{45}Ca$ uptake. Theophylline significantly increased insulin release from 2 tumours with a small stimulatory effect on the third. A depolarising concentration of $K^+$ enhanced insulin release from one tumour but this was not associated with an increase of $^{45}Ca$ uptake. Calcium antagonists, (verapamil, D-600 and trifluoroperazine) and calcium ionophores (A23187 and Br-X537A) failed to modify insulin release or $^{45}Ca$ uptake by each of the two tumours tested. Evaluation of $^{45}Ca$ efflux from one tumour confirmed the unresponsiveness to glucose, $K^+$, verapamil and A23187. Prolonged culture of 2 tumours for up to 16 days was associated with the gradual decline of insulin release to a steady output of 2–15 ng $24\,h^{-1}$. Addition of verapamil to the cultures inhibited insulin output from one tumour, but mannoheptulose or diazoxide were without effect. The results indicate that inappropriate insulin release from these 3 benign medullary-type insulinomas is associated with disturbances in the regulation of transmembrane $Ca^{2+}$ fluxes.

During the past decade, considerable evidence has accumulated concerning the role of metabolic and ionic events in the regulation of pancreatic B-cell function and insulin secretion (Hellman *et al.*, 1979; Wollheim & Sharp, 1981; Malaisse, 1983; Henquin & Meissner, 1984). Numerous studies with rodent islets indicate that nutrient secretogogues such as glucose are metabolised by the pancreatic B-cells leading to an increase in cytoplasmic $Ca^{2+}$ concentration and the discharge of insulin by exocytosis. Contributions to cytoplasmic $Ca^{2+}$ under these circumstances may include displacement of $Ca^{2+}$ from intracellular sites such as mitochondria and endoplasmic reticulum, inhibition of $Ca^{2+}$ outward transport across the plasma membrane and stimulation of $Ca^{2+}$ entry into the B-cell. Several studies using human islets support this mechanism (Ashcroft *et al.*, 1971; Henriksson *et al.*, 1978; Andersson & Hellerstrom, 1980; Grant *et al.*, 1980; Jahr *et al.*, 1983; Harrison *et al.*, 1985), but analogous studies evaluating defective regulation of insulin secretion by human insulinomas have not been performed.

Insulin-secreting tumours (insulinomas) of the pancreas represent the most common type of enteropancreatic endocrine cancer in man which without clinical intervention can result in debilitation and premature death (Frerichs & Creutzfeldt, 1976; Marks & Rose, 1981; Friesen, 1982; Comi *et al.*, 1986). Thus despite the relatively low incidence of the disease (1–2 recognised cases per million of the population per annum), the diagnosis and treatment of these heterologous tumours have attracted considerable attention from pathologists and clinicians. In the present study, we report the functional and morphological characterisation of three human benign medullary-type insulinomas. In addition, we have used small pieces of each tumour maintained in tissue culture for *in vitro* studies on the regulation of transmembrane $Ca^{2+}$ fluxes, and evaluation of both the acute and long term effects of nutrients and drugs on insulin release.

Correspondence: P.R. Flatt.
Received 2 March 1987; and in revised form, 26 May 1987.

### Materials and methods

### Clinical histories

*Case I* The first tumour was obtained from a 60 year old woman who presented with a 3 year history of 'odd turns' associated with a sense of unreality. Fasting for 36 h coupled with moderate exercise produced a fall in plasma glucose concentrations to 1.0 mM, associated with pallor and a feeling of tiredness and mild disorientation. Plasma C-peptide concentrations were consistently inappropriately elevated (1.2–2.5 $\mu g\,l^{-1}$) during hypoglycaemic episodes. Plasma insulin concentrations were 5.0–10.0 $mU\,l^{-1}$. At operation, a 0.8 cm diameter tumour was removed from the head of the pancreas. The patient has remained well since operation with no further hypoglycaemic episodes.

*Case II* The second tumour was obtained from a 45 year old woman who presented with a 6 year history of 'fits' for which she had received treatment with anticonvulsant drugs with limited success. Fasting hypoglycaemia (1.4–1.8 mM) with inappropriately high plasma insulin and C-peptide concentrations (9.6–11.3 $mU\,l^{-1}$ and 2.3–2.8 $\mu g\,l^{-1}$, respectively) were demonstrated on several occasions once the correct diagnosis had been suspected. A 1.3 cm diameter tumour was removed from the body of the pancreas at operation. The patient has since remained well with no recurrence of hypoglycaemic symptoms.

*Case III* The third tumour was obtained from a 15 year old girl who presented with a 12 month history of recurrent episodes of drowsiness associated with hypoglycaemia (plasma glucose 1.0–2.0 mM). After an overnight fast on 2 consecutive days in hospital, she became drowsy and plasma glucose concentrations fell to 1.9–2.2 mM. Insulin and C-peptide concentrations at this time were 15.3–23.3 $mU\,l^{-1}$ and 3.5–4.1 $\mu g\,l^{-1}$, respectively. At operation, a small tumour was removed from the tail of the pancreas. The patient has remained well since operation and there have been no further episodes of hypoglycaemia.

### Special investigations

In addition to measurements of plasma glucose, insulin and C-peptide concentrations in the fed and fasted states, various radiographic and electrophysiological tests were performed. None provided any additional clinically useful information. In Cases I and II, glucose tolerance tests were non-contributory. Oral leucine, i.v. tolbutamide and glucagon tests were also uninformative, and in particular failed to reveal hyperinsulinaemic responses observed in some patients with insulinoma. These tests were not performed in Case III.

In vitro *investigations*

*Preparation and culture of tumour pieces* Immediately after removal at operation, tumours were placed in tissue culture medium RPMI-1640 (Gibco Europe Ltd., Paisley, UK) containing 11.1 mM glucose, 10% foetal calf serum with added antibiotics (100 U ml$^{-1}$ penicillin and 0.1 mg ml$^{-1}$ streptomycin; Gibco Europe Ltd., Paisley, UK). The tumours were transported to the laboratory within 90 min of removal. Using a sterile scalpel blade, the tumours were chopped into small pieces (25–120 pieces of ~100 µg) and then maintained in tissue culture for 3 days at 37°C in a humidified atmosphere of 5% $CO_2$ in air prior to *in vitro* tests. Individual tumour pieces were used for only one experiment as described below.

*Acute studies of insulin release and $^{45}Ca$ uptake* Acute studies of insulin release and $^{45}Ca$ uptake were conducted using 3 day cultured pieces of all three tumours. After rinsing in serum-free culture medium, groups of 2 pieces of tumour were incubated for 60 min at 37°C in a modified Krebs–Ringer bicarbonate buffer (pH 7.4), containing 20 mM N-2-hydroxyethyl-piperazine-N′-2-ethanesulfonic acid (HEPES), 115 mM NaCl, 24 mM $NaHCO_3$, 4.7 mM KCl, 2.56 mM $^{45}CaCl_2$ (7.8 Ci mol$^{-1}$; Amersham International Ltd., Amersham, UK) 1.2 mM $KH_2PO_4$, 1.2 mM $MgSO_4$ and 5 mg ml$^{-1}$ bovine serum albumin. Glucose and the following nutrients and drugs were added at the concentrations given in Tables I–III: theophylline, glyceraldehyde, mannoheptulose, $K^+$, diazoxide, verapamil, D-600 (Hoechst, Milton Keynes, UK), trifluoroperazine (Smith, Kline and French Laboratories Ltd., Welwyn Garden City, UK), A23187 (Calbiochem Ltd., San Diego, USA) and Br-X537A (Hoffman LaRoche and Company Ltd., Basel, Switzerland). After 60 min incubation, aliquots were taken and stored at −20°C for insulin assay. The tumour pieces were transferred and washed for 60 min at 1°C with 5 ml Tris buffer containing 2 mM $LaCl_3$ to remove extracellular and superficial $^{45}Ca$ prior to measurement of intracellular $^{45}Ca$ content (Hellman, 1978). Previous studies have shown that the $^{45}Ca$ taken up by connective tissue surrounding islet cells is removed by $La^{3+}$-washing (Flatt & Swanson–Flatt, 1985). After freeze-drying overnight and weighing, the tumour pieces were dissolved in 100 µl Soluene (Packard Instruments Ltd., Warrenville, USA). After addition of 5 ml Instafluor (Packard), tumour radioactivity was analysed by liquid scintillation spectrometry. Samples of the labelled incubation medium (5 µl) were used as external standards in the counting procedure. In the tables, the tumour content of $^{45}Ca$ is expressed in terms of millimoles of calcium with the same specific radioactivity as that of the incubation medium.

*Studies of $^{45}Ca$ efflux* In the case of tumour III, there was a sufficient number of tumour pieces to enable evaluation of $^{45}Ca$ efflux in addition to the other parameters. For this purpose, groups of 2 pieces of tumour (previously cultured for 3 days) were briefly rinsed and then loaded with $^{45}Ca$ during incubation for 90 min in 100 µl of the modified Krebs–Ringer bicarbonate buffer supplemented with 20 mM glucose and 2.56 mM $^{45}Ca$ (360 Ci mol$^{-1}$). After two 5 min washes in nonradioactive medium containing 3 mM glucose, groups of 2 tumour pieces were transferred to 10 µl chambers and perifused for 90 min at a constant rate of ~40 µl min$^{-1}$ (Flatt *et al.*, 1980). The perifusate was collected over successive periods of 1 or 5 min, with inclusion of 16.7 mM glucose, 25 mM $K^+$, 50 µM verapamil or 20 µM A23187 in the buffer from 35–75 min as indicated in Figures 2 and 3. Samples of the perifusate (15 µl) were mixed with 2 ml Picofluor-15 (Packard Instruments Ltd., Warrenville, USA) and analysed for radioactivity by liquid scintillation counting. In each individual experiment, the $^{45}Ca$ efflux rate (c.p.m. min$^{-1}$) was expressed as a percentage of the mean value observed in the same experiment between the 31st and 36th minutes of perifusion.

*Long term studies of insulin release* Long term studies of insulin release were performed using pieces of tumour I and tumour III. After 3 days culture, groups of 2 tumour pieces, matched for equal size, were cultured in the same type of medium for an extended period of up to 16 days. Diazoxide (0.54 mM; Glaxo Group Research, Ware, UK), verapamil (50 µM; Abbot Laboratories Ltd., Queensborough, UK) or mannoheptulose (15 mM; Sigma Chemical Company, Poole, UK) were included in the culture media from days 3–4 onwards as indicated in Figures 1 and 2. Incubations were performed at 37°C (pH 7.4) in a gas phase consisting of 5% $CO_2$ in humidified air. The media were changed at 2–5 day intervals. In the case of tumour I, the media were removed at 4, 7, 11 and 14 days. For tumour III, the media were removed at 3, 6, 8, 13 and 16 days. Aliquots of culture media removed at these times were stored at −20°C for insulin assay.

*Assays*

Plasma glucose was measured by an automated glucose oxidase procedure (Stevens, 1971). Insulin was determined by dextran-charcoal radioimmunoassay (Flatt & Bailey, 1981) using guinea pig anti-porcine insulin antiserum, $^{125}I$-bovine insulin tracer (Amersham International, Amersham, UK) and human insulin standard (23.5 IU mg$^{-1}$; WHO International Laboratory for Biological Standards, London, UK). C-peptide was measured by double antibody radioimmunoassay (Hampton & Marks, 1979) using reagents supplied by Guildhay Antisera (University of Surrey, Guildford, UK). Parallelism was demonstrated between the insulin and C-peptide standard curves and serial dilutions of either plasma or samples arising from the *in vitro* investigations. All analyses were performed within 2 weeks of sample collection.

*Histology and immunocytochemistry*

Several small pieces of each tumour were fixed for a minimum of 24 h in neutral buffered formalin, dehydrated through graded ethanols, cleared in toluene and embedded in paraffin wax for histological and immunocytochemical investigation. Rehydrated paraffin sections (5 µm) were stained with haematoxylin and eosin, or immunostained by the indirect immunoperoxidase technique using guinea pig anti-porcine insulin antiserum (GPB3; PRF/SKS-F), guinea pig anti-porcine glucagon antiserum (GPC4; Flatt & Swanston–Flatt, 1981) and affinity purified donkey anti-guinea pig immunoglobulin G conjugated to horse radish peroxidase (Guildhay Antisera). Somatostatin and PP were immunostained by the unlabelled peroxidase anti-peroxidase (PAP) technique (Sternberger, 1979) using the following antisera: rabbit anti-cyclic somatostatin (GR21A; Guildhay Antisera), rabbit and anti-bovine pancreatic polypeptide (GR39PD; Guildhay Antisera), donkey and anti-rabbit immunoglobulin G (Guildhay Antisera) and rabbit PAP complex (Dakopatts, Glostrup, Denmark). Peroxidase activity was visualised using 3,3′-diaminobenzidine and sections were lightly counterstained with Harris' haematoxylin. Control sections were treated with normal serum instead of the hormone antisera.

*Statistical analysis*

Values are presented as mean±s.e.m. Statistical evaluation was performed using Student's paired and unpaired *t*-test. Differences were considered to be significant for $P<0.05$.

### Results

### *Histology and immunocytochemistry*

At surgery, an adenoma was removed from the pancreas of each of the patients. This was located in the head, body and tail of the pancreas for patients I, II and III respectively. Tumour metastases in the liver or other organs were not detected, and all patients were still free of hypoglycaemic symptoms 3–4 years after operation.

Immunocytochemical staining of the 3 tumours (Figure 1) confirmed the presence of abundant insulin-containing B-cells with no demonstrable staining for glucagon, somatostatin or pancreatic polypeptide. The histological arrangement of cells in each tumour was medullary-type (group B according to classification by Berger *et al.,* 1983) with positive insulin immunostaining cells distributed among negative cells with no polarisation. Tumour I was characterised by an abundant mature fibrous matrix; tumour II was highly vascularised with evidence of focal mineralisation, and tumour III was strongly immunostaining and contained moderate amounts of fibrous connective tissue.

### In vitro *investigations*

*Acute studies of insulin release and $^{45}Ca$ uptake* As shown in Tables I–III, pieces of tumour from the 3 patients released 11–158 mg insulin $kg^{-1}$ dry wt during 60 min incubations with the concomitant intracellular uptake of 2–47 mmol $^{45}Ca\,kg^{-1}$. Addition of glucose alone or in combination with glyceraldehyde, mannoheptulose or diazoxide did not modify insulin release or $^{45}Ca$ uptake by any of the tumours, with the exception of a small increase of $^{45}Ca$ uptake by pieces of tumour II exposed to 5.6 mM glucose. Addition of theophylline, increased insulin release from tumours II and III by 56–77% without affecting $^{45}Ca$ uptake. A similar tendency was observed with pieces of tumour I (50% increase), although this did not achieve statistical significance because of the scatter of individual values. However, 25 mM $K^+$ stimulated insulin release by 57% from tumour I without affecting $^{45}Ca$ uptake. As indicated by Tables I and III, the calcium antagonists verapamil, D-600 and trifluoroperazine, and the calcium ionophores A23187 and Br-X537A failed to modify insulin release or $^{45}Ca$ uptake by pieces of tumours I and III. These agents were not evaluated using tumour II due to shortage of tissue.

*Studies of $^{45}Ca$ efflux* The effects of glucose, 25 mM $K^+$, verapamil and A23187 on $^{45}Ca$ efflux following tissue preloading with $^{45}Ca$ were examined using pieces of tumour III. As shown in Figures 2 and 3, the $^{45}Ca$ efflux rate from tumour pieces was little affected by addition or removal of these agents from the perifusion media. This unresponsiveness is consistent with the results of acute studies of $^{45}Ca$ uptake (Table III).

*Long term studies of insulin release* Prolongation of culture for an extended period of 14–16 days was associated with a gradual decline of insulin release to steady outputs of ~15 ng insulin $24\,h^{-1}$ from tumour I (Figure 4) and 2 ng insulin $24\,h^{-1}$ from tumour III (Figure 5) by 4–7 days and 8–13 days, respectively. This progressive decline of insulin

**Table I** $^{45}Ca$ uptake and insulin release by tumour pieces during acute incubations: Tumour I – located in the head of the pancreas of a 60 year old woman

| *Additions (mM)* | *Glucose (mM)* | *$^{45}Ca$ uptake ($mmol\,kg^{-1}\,h^{-1}$)* | *Insulin release ($mg\,kg^{-1}\,h^{-1}$)* |
|---|---|---|---|
| *Nutrients and drugs* | | | |
| None | 0 | 2.08±0.14 | 22.94±4.20 |
| None | 5.6 | 2.03±0.22 | 23.02±3.20 |
| None | 16.7 | 2.71±0.11 | 19.32±3.40 |
| Theophylline (5) | 16.7 | 2.33±0.36 | 38.90±16.22 |
| Glyceraldehyde (10) | 5.6 | 3.19±0.82 | 27.90±2.53 |
| Mannoheptulose (15) | 5.6 | 2.28±0.27 | 38.92±9.20 |
| KCl (25) | 5.6 | 2.07±0.18 | 54.14±7.20[a] |
| Diazoxide (0.54) | 5.6 | 2.14±0.18 | 29.68±7.34 |
| *Calcium antagonists* | | | |
| Verapamil (0.05) | 5.6 | 2.67±0.44 | 33.88±6.77 |
| D-600 (0.02) | 5.6 | 2.02±0.15 | 29.05±3.86 |
| Trifluoroperazine (0.02) | 5.6 | 2.09±0.20 | 29.95±4.31 |
| *Calcium ionophores* | | | |
| A23187 (0.02) | 5.6 | 3.42±1.67 | 37.97±9.02 |
| X537A (25 $\mu g\,ml^{-1}$) | 5.6 | 1.85±0.25 | 34.76±11.01 |

Groups of 3-day cultured tumour pieces were incubated for 60 min in buffer containing 2.56 mM $^{45}Ca$ ($7.8\,Ci\,mol^{-1}$), 0, 5.6 or 16.7 mM glucose, and the nutrients or drugs as indicated. Radioactivity in tumour pieces was measured after subsequent washing for 60 min with 2 mM $LaCl_3$ at 1°C to remove extracellular and superficially bound $^{45}Ca$. The amount of intracellular $^{45}Ca$ is expressed as mmol of calcium, assuming the same specific radioactivity as in the incubation buffer. Values are mean ±s.e.m. of 3–4 observations. [a]$P < 0.05$ compared with 5.6 mM glucose.

**Figure 1** Immunohistochemical staining for insulin in sections of human tumour. Tumour I (left panel): clumps of strongly immunostaining cells are closely associated with vascular spaces. Tumour II (middle panel): large numbers of immunostaining cells are interspersed with vascular tissue. Tumour III (right panel): nests of intensely immunostaining cells are mixed with connective tissue (×313).

**Table II** $^{45}Ca$ uptake and insulin release by tumour pieces during acute incubations: Tumour II – located in the body of the pancreas of a 45 year old woman

| *Additions (mM)* | *Glucose (mM)* | *$^{45}Ca$ uptake (mmol kg$^{-1}$ h$^{-1}$)* | *Insulin release mg kg$^{-1}$ h$^{-1}$)* |
|---|---|---|---|
| *Nutrients and drugs* | | | |
| None | 0 | 21.29±4.10 | 24.94±3.31 |
| None | 5.6 | 37.70±1.48[a] | 44.44±13.53 |
| None | 16.7 | 23.99±8.57 | 36.26±4.48 |
| Theophylline (5) | 16.7 | 31.84±10.36 | 158.41±52.30[ab] |
| Glyceraldehyde (10) | 5.6 | 47.44±15.50 | 70.35±22.92 |
| Mannoheptulose (15) | 5.6 | 29.00±8.27 | 26.02±2.47 |
| KCl (25) | 5.6 | 36.67±9.58 | 34.69±0.89 |
| Diazoxide (0.54) | 5.6 | 24.29±6.76 | 47.55±16.08 |

Groups of 3-day cultured tumour pieces were incubated for 60 min in buffer containing 2.56 mM $^{45}Ca$ (7.8 Ci mol$^{-1}$), 0, 5.6 or 16.7 mM glucose, and the nutrients or drugs as indicated. Radioactivity in tumour pieces was measured after subsequent washing for 60 min with 2 mM $LaCl_3$ at 1°C to remove extracellular and superficially bound $^{45}Ca$. The amount of intracellular $^{45}Ca$ is expressed as mmol of calcium, assuming the same specific radioactivity as in the incubation buffer. Values are mean±s.e.m. of 3–4 observations. [a]$P<0.001$ compared with 0 mM glucose; [b]$P<0.05$ compared with 16.7 mM glucose.

**Table III** $^{45}Ca$ uptake and insulin release by tumour pieces during acute incubations: Tumour III – located in the tail of the pancreas of a 15 year old girl

| *Additions (mM)* | *Glucose (mM)* | *$^{45}Ca$ uptake (mmol kg$^{-1}$ h$^{-1}$)* | *Insulin release (mg kg$^{-1}$ h$^{-1}$)* |
|---|---|---|---|
| *Nutrients and drugs* | | | |
| None | 0 | 6.37±0.58 | 20.12±5.87 |
| None | 5.6 | 5.68±0.59 | 17.95±2.67 |
| None | 16.7 | 5.00±0.32 | 15.34±1.07 |
| Theophylline (5) | 16.7 | 6.33±0.71 | 35.13±2.26[a] |
| Glyceraldehyde (10) | 5.6 | 5.41±0.13 | 14.39±2.10 |
| Mannoheptulose (15) | 5.6 | 6.30±0.79 | 18.90±3.61 |
| KCl (25) | 5.6 | 8.52±2.58 | 32.95±7.46 |
| Diazoxide (0.54) | 5.6 | 6.67±0.53 | 11.66±0.97 |
| *Calcium antagonists* | | | |
| Verapamil (0.05) | 5.6 | 7.34±0.53 | 11.99±0.94 |
| D-600 (0.02) | 5.6 | 5.66±0.54 | 15.74±4.83 |
| Trifluoroperazine (0.02) | 5.6 | 7.03±0.56 | 18.92±5.03 |
| *Calcium ionophores* | | | |
| A23187 (0.02) | 5.6 | 6.41±0.38 | 16.74±2.56 |
| X537A (25 μg ml$^{-1}$) | 5.6 | 8.45±1.23 | 16.50±1.72 |

Groups of 3-day cultured tumour pieces were incubated for 60 min in buffer containing 2.56 mM $^{45}Ca$ (7.8 Ci mol$^{-1}$), 0, 5.6 or 16.7 mM glucose, and the nutrients or drugs as indicated. Radioactivity in tumour pieces was measured after subsequent washing for 60 min with 2 mM $LaCl_3$ at 1°C to remove extracellular and superficially bound $^{45}Ca$. The amount of intracellular $^{45}Ca$ is expressed as mmol of calcium, assuming the same specific radioactivity as in the incubation buffer. Values are mean ±s.e.m. of 3–4 observations. [a]$P<0.05$ compared with 5.6 mM glucose.

output is commonly observed during the long term culture of insulin-secreting cells (Andersson & Hellerstrom, 1980). Addition of verapamil to the culture medium inhibited insulin output from pieces of tumour III. In contrast, diazoxide and mannoheptulose did not affect insulin output from tumour III, and neither drug affected tumour I. The demonstration of long term effects of verapamil on tumour III contrasts with the inability of the drug to acutely depress insulin release (Table III). This delayed action is suggestive of a non-specific effect, possibly on cellular insulin stores (Leinweber & Schatz, 1982).

**Figure 2** Effects of glucose and $K^+$ on $^{45}Ca$ efflux from pieces of tumour III – located in the tail of the pancreas of a 15 year old girl. Experiments were performed in the parallel channels of a perifusion apparatus with 3-day cultured tumour pieces loaded for 90 min with 2.56 mM $^{45}Ca$ (360 Ci mol$^{-1}$) in the presence of 20 mM glucose. The tumour pieces were perifused with buffer containing 3 mM glucose, with exposure to 16.7 mM glucose or 25 mM $K^+$ during the period from 35–70 min, as indicated by the horizontal bar. Filled symbols signify tumour pieces exposed to glucose (●) or $K^+$ (■), and open symbols refer to control tumour pieces (○). Values are mean ±s.e.m. of 3 individual experiments. $^{45}Ca$ efflux was expressed as a percentage of the mean value observed in the same experiment between the 31st and 36th min of perifusion.

**Figure 3** Effects of verapamil and A23187 on $^{45}Ca$ efflux from pieces of tumour III – located in the tail of the pancreas of a 15 year old girl. Experiments were performed in the parallel channels of a perifusion apparatus with 3-day cultured tumour pieces loaded for 90 min with 2.56 mM $^{45}Ca$ (360 Ci mol$^{-1}$) in the presence of 20 mM glucose. The tumour pieces were perifused with buffer containing 3 mM glucose, with exposure to 50 μM verapamil or 20 μM A23187 during the period from 35–70 min, as indicated by the horizontal bar. Filled symbols signify tumour pieces exposed to verapamil (■) or A23187 (●), and open symbols refer to control tumour pieces (○). Values are mean ±s.e.m. of 3 individual experiments. $^{45}Ca$ efflux was expressed as a percentage of the mean value observed in the same experiment between the 31st and 36th min of perifusion.

### Discussion

The study of insulin-secreting tumours in man has received much attention from the morphological, ultrastructural, diagnostic and therapeutic viewpoints (Frerichs & Creutzfeldt, 1976; Marks & Rose, 1981; Friesen, 1982; Comi *et al.*, 1986). However, little attention has been paid to biochemistry of insulin secreting tumours in relation to the underlying secretory defect. Thus, studies performed to date

**Figure 4** Long term studies in insulin release during culture of pieces of tumour I – located in the head of the pancreas of a 60 year old woman. After 3 days preliminary culture in RPMI-1640 containing 11.1 mM glucose and 10% foetal calf serum, groups of tumour pieces were cultured in the same type of medium for 14 days. Diazoxide (0.54 mM) or verapamil (50 $\mu$m) were included as appropriate in the culture media from 4 days onwards as indicated. The media were changed at 3–4 day intervals. Values are mean ±s.e.m. of 4 observations; □ control; ▨ Diazoxide; ■ Verapamil.

have concentrated on the molecular forms of insulin-like substances produced and mechanisms for insulin degradation in these cells. The major reason for the paucity of information is the sporadic incidcnce of the disease, and the fact that surgical treatment of such patients is restricted to specialised centres. The present study has made a detailed investigation of the regulation of insulin release and trans-membrane calcium fluxes from pieces of three human insulinomas obtained at operation. Advantage was taken of tissue culture to counter effects attributable to prior hypoglycaemia, and the unavoidable trauma of surgery and laboratory tissue preparation. This approach also enabled long-term studies of insulin release and ensured that a maximum amount of information could be gained from each tumour.

Although the tumours were derived from distinctly different regions of the pancreas of patients with a considerable age span, there were notable similarities of tumour morphology. Each was classified as a benign adenoma comprising abundant insulin-staining B-cells with no demonstable staining for other islet cell hormones including glucagon, somatostatin and pancreatic polypeptide. Furthermore, the histological arrangement of the cells indicates classification as a medullary-type tumour (group B) with positive insulin immunostaining cells diffusely scattered throughout the tumour with no polarisation (Berger *et al.*, 1983). Compared with the *in vivo* functional characteristics of the alternative trabecular-type (group A) insulinomas, tumours such as those evaluated in the present study have been proposed to display marked unresponsiveness to modulators of insulin release (Berger *et al.*, 1983). The present results of acute and long term *in vitro* tests of insulin release from each tumour in response to various nutrients and drugs fully supports this view.

In the present study, it was not feasible to perform parallel control experiments of insulin release and $^{45}Ca$ fluxes by human pancreatic islets. However, the few previous reports in the literature indicate that knowledge gained from studies of insulin secretion using rodent islets is almost entirely applicable to the regulation of insulin release in man (Ashcroft *et al.*, 1971; Andersson & Hellerstrom, 1980;

**Figure 5** Long term studies of insulin release during culture of pieces of tumour III – located in the tail of the pancreas of a 15 year old girl. After 3 days preliminary culture in RPMI-1640 containing 11.1 mM glucose and 10% foetal calf serum, groups of tumour pieces were cultured in the same type of medium for 16 days. Diazoxide (0.54 mM). mannoheptulose (15 $\mu$m) or verapamil (50 $\mu$M) were included as appropriate in the culture media from 3 days onwards as indicated. The media were changed at 3–5 day intervals. Values are mean ±s.e.m. of 4 observations. *$P<0.05$ compared with control culture medium; □ control, ▨ Diazoxide; ▩ Mannoheptulose; ■ Verapamil.

Henriksson et al., 1978; Grant et al., 1980; Jahr et al., 1983; Harrison et al., 1985). These studies have not only confirmed the substrate-specificity and the role of metabolism for nutrient-induced insulin release but have also highlighted the role of intracellular messengers such as $Ca^{2+}$, cyclic AMP and protein kinases in B-cell stimulus-secretion coupling. The ineffectiveness of the majority of nutrients and drugs tested by acute incubation, perifusion or long term culture in the present study may be cautiously interpreted therefore in terms of the defect(s) exhibited by the insulinoma cells. It is not possible to disregard the individual nature of each tumour, but several general conclusions can be drawn from the results obtained. Thus, none of the tumours responded to glucose or glyceraldehyde with stimulated $^{45}Ca$ uptake and insulin release. Furthermore, the metabolic inhibitor mannoheptulose was also without effect. Additional evidence for defective insulin release and abnormal cellular $Ca^{2+}$ metabolism concerns the virtual ineffectiveness of a depolarising concentration of $K^+$ and of diazoxide which are believed to exert stimulatory and inhibitory effects respectively on the opening of voltage-dependent $Ca^{2+}$ channels in the B-cell (Hellman et al., 1979; Wollheim & Sharp, 1981; Henquin & Meissner, 1984). Two established blockers of this $Ca^{2+}$ channel (verapamil and D-600) were also ineffective, as was the intracellular inhibitor of $Ca^{2+}$-calmodulin, trifluoroperazine. These observations together with insensitivity to the $Ca^{2+}$ ionophores, A23187 and Br-X537A, indicate marked irregularities in the regulation of transmembrane $Ca^{2+}$ fluxes and insulin release by these tumour cells. Interestingly, this is not the only observation indicating either lack of effect of calcium ionophores or general disturbances of $Ca^{2+}$ metabolism in cancer cells (Cittadini et al., 1981; Durham & Walton, 1982; Ralph, 1983).

Despite the failure of the tumours to respond as normal insulin secreting cells to nutrients, calcium antagonists and calcium ionophores, all three tumours exhibited evidence of stimulated insulin release in the presence of theophylline. This drug is an established inhibitor of cyclic AMP phosphodiesterase and potentiates insulin secretion through elevation of cyclic AMP without affecting intracellular $Ca^{2+}$ (Roseman & Abrahamsson, 1985). This suggests that these tumours may be responsive to agents modulating insulin secretion through the adenylate cyclase-cyclic AMP system (see Lins & Effendic, 1979), but as demonstrated by Veroni and colleagues (1980) this may not correlate with the apparent responsiveness of such tumours *in vivo*.

In conclusion, the present study has made a detailed investigation of the regulation of transmembrane $Ca^{2+}$ fluxes and insulin release in three benign functional medullary-type insulinomas obtained at surgery. The results suggest that inappropriate secretion of insulin from these tumours is associated with, and possibly stems from marked abnormalities in the regulation of voltage-gated $Ca^{2+}$ channels in the B-cell plasma membrane.

These studies were supported by a grant from the Cancer Research Campaign (SP 1630). We are grateful to Mr P.S. Boulter and Professor I. McColl for assistance in obtaining the tumours.

### References

ANDERSSON, A. & HELLERSTROM, C. (1980). Explant culture: Pancreatic islets. In *Methods in Cell Biology*, **Vol. 21B**, p. 135. Academic Press: New York.

ASHCROFT, S.J.H., BASSETT, J.M. & RANDLE, P.J. (1971). Isolation of human pancreatic islets capable of releasing insulin and metabolising glucose *in vitro*. *Lancet*, **i**, 888.

BERGER, M., BORDI, C., CUPPERS, H.-J. & 6 others (1983). Functional and morphological characterisation of human insulinomas. *Diabetes*, **32**, 921.

CITTADINI, A., BOSSI, D., DANI, A.M., CALVIELLO, G., WOLF, F. & TERRANOVA, T. (1981). Lack of effect of the $Ca^{2+}$ ionophore A23187 on tumour cells. *Biochim. Biophys. Acta*, **645**, 177.

COMI, R.J., GORDEN, P., DOPPMAN, J.L. & NORTON, J.A. (1986). Insulinoma. In *The Exocrine Pancreas: Biology, Pathobiology and Diseases*, Go, V.L.W. (ed) p. 745. Raven Press: New York.

DURHAM, A.C.H. & WALTON, J.M. (1982). Calcium ions and the control of proliferation in normal and cancer cells. *Biosci. Rep.*, **2**, 15.

FLATT, P.R., & BAILEY, C.J. (1981). Abnormal plasma glucose and insulin responses in heterozygous (ob/+) mice. *Diabetologia*, **20**, 573.

FLATT, P.R., BERGGREN, P.-O., GYLFE, E. & HELLMAN, B. (1980). Calcium and pancreatic B-cell function. IX. Demonstration of lanthanide-induced inhibition of insulin secretion independent of modifications in transmembrane $Ca^{2+}$ fluxes. *Endocrinology*, **107**, 1007.

FLATT, P.R. & SWANSTON-FLATT, S.K. (1981). Stimulation of antiglucagon antibodies in rabbits and guinea pigs using a glucagon-carbodiimide-albumin conjugate. *Endocrinologia experimentalis*, **15**, 3.

FLATT, P.R. & SWANSTON-FLATT, S.K. (1985). Effects of decapsulation on superficial and intracellular $^{45}Ca$ uptake by isolated mouse pancreatic islets. *Biomed. Res.*, **4**, 557.

FRERICHS, H. & CREUTZFELDT, W. (1976). Hypoglycaemia: Insulin-secreting tumours. *Clin. Endocrinol. Metab.*, **5**, 747.

FRIESEN, S.R. (1982). Tumours of the endocrine pancreas. *New Engl. J. Med.*, **306**, 580.

GRANT, A.M., CHRISTIE, M.R. & ASHCROFT, S.J.H. (1980). Insulin release from human pancreatic islets *in vitro*. *Diabetologia*, **19**, 114.

HAMPTON, S.M. & MARKS, V. (1979). Development, validation and use of a human C-peptide radioimmunoassay. *Diabetologia*, **17**, 24.

HARRISON, D.E., CHRISTIE, M.R. & GRAY, D.W.R. (1985). Properties of isolated human islets of Langerhans: Insulin secretion, glucose oxidation and protein phosphorylation. *Diabetologia*, **28**, 99.

HELLMAN, B. (1978). Calcium and pancreatic B-cell function: Validity of the $La^{3+}$-wash technique for discriminating between superficial and intracellular $^{45}Ca$. *Biochim. Biophys. Acta*, **540**, 534.

HELLMAN, B., ANDERSSON, T., BERGGREN, P.-O., FLATT, P.R., GYLFE, E. & KOHNERT, K.D. (1979). The role of calcium in insulin secretion. In *Hormones and Cell Regulation*, **Vol. 3** Dumont, J. & Nunez, J. (ed) p. 69. Elsevier: Amsterdam.

HENQUIN, J.C. & MEISSNER, H.P. (1984). Significance of ionic fluxes and changes in membrane potential for stimulus-secretion coupling in pancreatic B-cells. *Experientia*, **40**, 1043.

HENRIKSSON, C., CLAES, G., GYLFE, E., HELLMAN, B. & ZETTERGREN, L. (1978). Collagenase isolation and $^{45}Ca$ efflux studies of human islets of Langerhans. *Eur. Surg. Res.*, **10**, 343.

JAHR, H., RATZMANN, K.-P., BECKERT, R., BESCH, W. & HAHN, H.-J. (1983). Enhanced synthesis, storage and secretion of insulin in pancreatic islets derived from obese subjects. *Metabolism*, **32**, 1101.

LEINWEBER, K.P. & SCHATZ, H. (1982). Long-term and short-term effects of calcium, verapamil and diazoxide on biosynthesis and release of (pro-)insulin in isolated islets of rat pancreas. *Acta Endocrinologica*, **99**, 94.

LINS, P.-E. & EFENDIC, S. (1979). Responses of patients with insulinomas to stimulators and inhibitors of insulin release that have been linked with cyclic adenosine monophosphate. *Diabetes*, **28**, 190.

MALAISSE, W.J. (1983). Insulin release: The fuel concept. *Diab. Metab.*, **9**, 313.

MARKS, V. & ROSE, F.C. (1981). *Hypoglycaemia*, 2nd edn. Blackwell Scientific Publications: Oxford.

RALPH, R.K. (1983). Cyclic AMP, calcium and the control of cell growth. *FEBS Lett.*, **161**, 1.

RORSMAN, P. & ABRAHAMSSON, H. (1985). Cyclic AMP potentiates glucose-induced insulin release from mouse pancreatic islets without increasing cytosolic free $Ca^{2+}$. *Acta Physiol. Scand.*, **125**, 639.

STERNBERGER, L.A. (1979). *Immunocytochemistry*, 2nd edn. Wiley & Sons: New York.

STEVENS, J.F. (1971). Determination of glucose by an authomatic analyser. *Clin. Chim. Acta*, **32**, 199.

VERONI, M.C., MICHELANGELI, V.P., HEANEY, T.P., NG, K.W., PARTRIDGE, N.C. & LARKINS, R.G. (1981). Adenylate cyclase responsiveness of human insulinomas. *Horm. Metab. Res.*, **13**, 254.

WOLLHEIM, C.B. & SHARP, G.W.G. (1981). Regulation of insulin release by calcium. *Physiol. Rev.*, **61**, 914.